# Everybody's Page

**Published:** 1889-08-10

**Authors:** 


### Everybody's Page.

Lime water, or aerated water of some kind, renders milk more digestible.

A valuable deposit of kaolin, or porcelain clay, has been found in the Island of If, in Sweden.

Indian ink is made in some unknown way from burnt camphor. The secret is known only to the Chinese, and they refuse to reveal it.

A skilful cork-cutter can produce from 1,500 to 2,000 corks a day, his only tools being two sharp knives with broad blades. Machines have been brought out which turn out about 2,000 corks an hour, but they are useless for the cutting of the finer varieties.

In St. Louis they have a live barometer. It is a small tree toad, an animal very susceptible to changes of weather, shut in a glass tube. There is a little ladder for it to climb, and whenever the air grows moist, in anticipation of rain, it goes up to the top, coming down again when clear weather is at hand.

A German physician recommends salicylate of sodium as a cure for toothache. Ten grain doses, taken internally every half-hour till the pain goes away, afford relief for several days. It is specially useful in toothache connected with rheumatism. This treatment, except under medical supervision, is highly dangerous.

The phrase "A hair of the dog that bit you," though now confined to a symbolic and alcoholic interpretation, has an accurately canine origin. In the Caucasus it is still common for anyone who is bitten by a dog to lay a handful of hair, taken from the same animal's coat, upon the wound before cauterising and bandaging it. In some mystic way the hair is supposed to prevent untoward consequences.

Dr. Norman Kerr, who was first drawn to the study of inebriety as a disease while working as a temperance reformer, points out that certain periods of life are accompanied by a special craving for stimulants, which disappears along with the physiological circumstances that caused the disturbance of the system. The habit of drunkenness may also result from some accident, may indeed be a symptom of some obscure brain disease.

An American chemist has protested against the disuse of the old-fashioned infusions and decoctions of drugs in favour of the alcoholic preparations now in use. He admits that these are more agreeable, both to eye and palate, than the old-fashioned drugs, but doubts if in all cases they are equally efficacious. Indeed, he brings forward several instances in which a drug has gained credit when used as an infusion, and lost it when the alcoholic preparation was employed.

"Miranda," in the *Lady's Pictorial*, has made a thoroughly sensible and seasonable suggestion. Why should women during the hot weather imprison their hands in gloves? The custom is expensive, uncomfortable, and in a slight degree unhealthy, for the tight glove often prevents free circulation. If the hands were left uncovered they might acquire a coating of tan; but this is neither uncomfortable nor unbecoming; and it is one of the few luxuries within the reach of all. Gloves, as a protection from cold, are reasonable enough; but with regard to the imprisonment of the hands in hot weather, we feel inclined to echo the opinion of the negro soldier, who, when he was first promoted to the dignity of gloves and shoes, said, "Barracky for footy bad enuff; barracky for handy tooey bad—tooey bad."

Picrate of iron is said to be one of the best remedies for bleeding from the nose.

Two French chemists—Messieurs Hautefeuille and Perrey—have succeeded in making crystals which have all the appearance of fine emeralds.

The young lady who recently passed the examination of the Philadelphia College of Pharmacy, is called by her fellow-graduates their pharma-sister.

"So your papa's a doctor," said a well-meaning lady to a little girl. "I suppose he practices a good deal?" But the loyal daughter replied, indignantly, "No, he doesn't practice at all. He knows how to cure people."

Nutmegs and mace, which come from the same tree, the nutmeg being surrounded by mace, are characterised by a narcotic quality not unlike that of opium, though very much weaker.

A kind of tobacco grown near Smyrna has a special property of inducing irritation at the back of the throat, and it is probable that the mixture of this with Turkish tobacco, to give a more piquant flavour, is one cause of the complaints often made of throat disease resulting from the use of Turkish or Egyptian cigarettes.

Paraldelyde seems to be one of the most satisfactory hypnotics to be found. It is rash to say that anything of the kind is perfectly harmless; but in cases of insomnia or delirium, where sleep must be procured at any price, it has given satisfactory results, without the serious symptoms produced by morphia, chloral, or bromide of potassium.

The Brooklyn doctors are wise men, and know how to look after their own interests. There is a numerous class of patients who go to one doctor till his bill comes in, and then transfer their undesirable patronage to another. To prevent the success of these ingenious persons, the doctors in Brooklyn have formed a "Physicians' Protective Alliance," which is making a list of these unremunerative patients, so that the doctor can, if he chooses, refuse to attend them unless paid in advance.

The heathen Chinee, who is nothing if not economical, has an ingeniously simple method of eating his cake—or, rather in the Celestial translation, drinking his tea—and having it also. He makes the tea, drinks the delicate first infusion, and then dries the leaves again; packs them in those mysteriously-lettered boxes we know, and exports them to the Western barbarian, who, he has found out, likes his tea strong and stewed. It is true that the tannic acid apt to come out of the leaf in the second infusion may injure the British stomach. That is not John Chinaman's business; he knows the beauty and profit of the art of adulteration.

Mr. H. Sullivan Thomas has been lecturing the inhabitants of Madras on the mosquito, which he declares to be a blessing in disguise. This much-wronged insect devotes seven-eighths of its whole existence to the humble but necessary duties of a scavenger. During the twenty-one days of its larval existence it is only too happy to get into a pool of dirty water or any equally unpleasant locality and devour the contaminating matter. The larvæ are to be found in thousands in such places enthusiastically engaged in sanitary work. It is only in its brief existence in the winged state that it makes its presence felt in an obnoxious way; and Mr. Thomas was ungallant enough to say that it was the female mosquito that did all the biting.

